# Oxygen delivery and consumption in aging skeletal muscle: Insights from an electric analogy model of PO_2_ transients

**DOI:** 10.14814/phy2.70741

**Published:** 2026-01-23

**Authors:** Aleksander S. Golub, Roland N. Pittman, William H. Nugent, Bjorn K. Song

**Affiliations:** ^1^ Song Biotechnologies, LLC Baltimore Maryland USA; ^2^ Department of Physiology and Biophysics, Medical College of Virginia Virginia Commonwealth University Richmond Virginia USA

**Keywords:** aging muscle, circuit model, interstitial PO_2_, muscle respiration, PO_2_ transients, rat spinotrapezius

## Abstract

Mathematical models are essential for understanding oxygen transport and utilization during metabolic transitions. An electrical analogy concept proposed that exponential PO_2_ transients arise from interaction between oxygen storage capacitance and transport conductances, but lacked explicit circuit representation limiting quantitative predictions and experimental testing. We developed an explicit electrical circuit model with discrete resistive and capacitive components in physiologically defined topology to generate testable predictions for interstitial PO_2_ transition dynamics during rest‐work transitions in skeletal muscle. Circuit topology was constructed based on established physiological relationships in rat spinotrapezius muscle. The model equated oxygen partial pressure to voltage, oxygen flux to current, delivery and metabolic barriers to resistances, and tissue oxygen storage to capacitance. The model predicted that transition time constants should equal the product of capacitance and equivalent circuit resistance. Predictions were validated using interstitial PO_2_ measurements during rest‐work‐rest transitions. The model successfully predicted asymmetric transition kinetics, with time constant ratios matching steady‐state PO_2_ ratios. Application to young (3‐month) and old (23‐month) rats quantified age‐related changes: 2.5‐fold higher delivery resistance in old muscle with compensatory 5.4‐fold metabolic resistance reduction during exercise versus 3.1‐fold in young muscle. An explicit, validated electrical circuit model confirmed that PO_2_ transition kinetics are governed by capacitance‐resistance interactions and quantitatively separated delivery versus metabolic limitations in aging muscle.

## 1 INTRODUCTION

Mathematical models are essential for understanding the interactions between oxygen transport and utilization during both steady‐state and transient conditions.

In 1982, J. Piiper (1982) established an electrical analogy for tissue oxygen transport, mapping oxygen partial pressure to voltage, oxygen flux to current, and transport barriers to conductance (the reciprocal of resistance). He introduced perfusive and diffusive conductance and oxygen capacitance governing tissue O_2_ storage responses to PO_2_ changes. For abrupt increases in metabolic rate at exercise onset, Piiper proposed that exponential kinetics of PO_2_ and VO_2_ follow a time constant equal to the capacitance‐to‐conductance ratio. His electrical analogy represented the distributed properties of oxygen transport and consumption through conductance and capacitance, with the hypothesis that exponential transients arise from their interaction according to electrical circuit principles.

While Piiper established the conceptual framework, he did not construct an explicit circuit representation. We converted Piiper's concepts into an explicit circuit model with discrete components connected in physiologically defined topology. Arterial blood PO_2_ serves as the voltage source, while delivery resistance represents the diffusive barrier from capillaries to interstitial space. The interstitial compartment functions as the circuit node connecting delivery and consumption pathways, with metabolic resistance switching between resting and working values. Oxygen storage capacitance connects at the interstitial node, while oxygen conversion to water defines the circuit's zero potential. This resistor‐capacitor configuration enables the application of Kirchhoff's laws and Thévenin's theorem to generate quantitative predictions for transient dynamics, with additional advantages including well‐established graphical symbolism, mathematical methods for circuit analysis, and software simulation tools.

A critical assumption underlying transient predictions is that metabolic rate changes occur rapidly at exercise onset (Piiper, 1982). The interaction between tissue O_2_ capacitance and delivery resistance determines the transient time constant only if cellular respiration changes much faster than interstitial PO_2_ changes. Experimental evidence supports this assumption. Classic studies measuring mitochondrial respiration activation by rapid ADP concentration changes found responses fast enough (<1 s) to explain physiological transitions (Chance & Williams, 1955). Direct observations of mitochondrial activation in rat neurons during stimulation showed flavoprotein fluorescence increases with 0.7 s time constants (Vazquez et al., 2012). Measured time constants for rest‐to‐work transitions in spinotrapezius muscle are 14.8 s, with work‐to‐rest recovery requiring 40.9 s (Hirai et al., 2018, 2019). The notable difference between rapid mitochondrial activation and slower interstitial PO_2_ transients supports Piiper's hypothesis (Piiper, 1982) that exponential dynamics are governed by the interplay between oxygen delivery resistance, consumption rate, and tissue oxygen capacity.

Understanding oxygen transport mechanisms in skeletal muscle requires an experimental system enabling direct measurements of interstitial oxygen dynamics during metabolic transitions. The rat spinotrapezius muscle serves as a standard preparation for studying oxygen partial pressure and consumption rate during exercise of constant power (Bailey et al., 2000; Golub & Pittman, 2012; Gray, 1973; Nugent et al., 2016). This thin, planar muscle permits bio‐microscopy, temperature control, electrical stimulation, and compression experiments with minimal cardiovascular perturbation, making it well‐suited for microcirculatory oxygen research.

Phosphorescence quenching microscopy enables non‐invasive measurement of oxygen partial pressure at the microscopic level in microvascular and interstitial compartments, directly revealing oxygen transport and metabolic processes (Papkovsky & Dmitriev, 2018; Vanderkooi et al., 1987, 1991). The interstitial space between capillaries and muscle fibers functions as the circuit node where arterial PO_2_ undergoes potentiometric division determined by delivery and metabolic resistances. Interstitial PO_2_ measurements quantify this arterial PO_2_ division ratio during steady‐state rest and work in young and aged muscle (Golub et al., 2023; Hirai et al., 2018, 2019; Nugent et al., 2016; Wilson et al., 1988). During rest‐to‐work and work‐to‐rest transitions, interstitial PO_2_ dynamics reflect the interaction between resistances and oxygen storage capacitance, with transient time constants providing insight into the values of these circuit components that govern the exponential approach to new steady states (Behnke et al., 2002, 2009; Ferreira et al., 2005; Golub et al., 2023).

We measured VO_2_ directly by monitoring interstitial PO_2_ during rapid muscle compression with a pneumatic membrane. Supra‐systolic pressure stops blood flow and expels microvascular blood, creating oxygen disappearance curves. VO_2_ calculations derive from the initial slope of the PO_2_ curve multiplied by the tissue Bunsen O_2_ solubility coefficient. Differentiation of the entire curve determines the oxygen dependence of VO_2_ on PO_2_ (Golub et al., 2011). These experiments provided both the steady‐state PO_2_ and VO_2_ values and transient time constants needed to calculate circuit component values and test the model's predictions regarding the physical basis of oxygen dynamics.

These measurement capabilities enabled investigation of age‐related changes in oxygen transport mechanisms. Respiration in young and old muscle during rest and exercise depends on adequate oxygen supply to meet metabolic demand. With aging, both microvascular oxygen transport and mitochondrial function decline significantly, but the functional changes and their physiological manifestations remain poorly characterized (Behnke et al., 2005; McCullough et al., 2011; Nyberg & Jones, 2022; Russell et al., 2003). Aging studies in rat spinotrapezius muscle documented significant declines in capillary hemodynamics and microvascular PO_2_ in senescent animals (Landers‐Ramos & Prior, 2018; McCullough et al., 2011; Russell et al., 2003).

Age‐related muscle function decline has generated two major explanations. The vascular theory attributes decreased microvascular and interstitial PO_2_ and slower oxygen kinetics to impaired delivery from age‐related vascular changes (Muller‐Delp, 2016), including reduced feeding arterioles and decreased capillary density (Amara et al., 2007). The mitochondrial theory suggests slower oxygen kinetics reflect compensatory reductions in oxidative enzyme content associated with sarcopenia. Dysregulation of mitochondrial biogenesis in both myocytes and vascular cells creates complex interactions between vascular and metabolic limitations (Muller‐Delp, 2016).

Despite extensive research on vascular and mitochondrial aging, significant gaps remain in understanding how these mechanisms integrate to produce observed age‐related changes in oxygen dynamics. Most studies approach this problem from physiological or biochemical perspectives without explaining the physical basis of oxygen transients during activity transitions. We applied our circuit model to experimental data from young and old rat spinotrapezius muscle to calculate delivery and metabolic resistances, providing a quantitative assessment of age‐related functional changes in oxygen transport and consumption.

This study has three specific aims: (1) Develop an electrical circuit model for skeletal muscle oxygen transport by converting Piiper's conceptual framework into explicit circuit topology with discrete resistive and capacitive components, enabling systematic circuit analysis. (2) Validate the model by testing its prediction that interstitial PO_2_ transition kinetics arise from tissue oxygen storage capacitance interacting with the circuit's equivalent resistance. (3) Apply the model to quantify age‐related changes in oxygen delivery and metabolic regulation by calculating circuit parameters from experimental data in young and old rat spinotrapezius muscle, evaluating vascular versus mitochondrial mechanisms underlying functional decline.

## 2 METHODS

### 2.1 Electrical circuit model for PO_2_ and VO_2_ in muscle

The driving force represents the oxygen partial pressure difference from arterial blood (PO_2_ = *P*_a_) to mitochondria (PO_2_ = 0). The oxygen flux VO_2_ represents O_2_ molecules being converted to water per second. So, setting the “sink” reference point at zero PO_2_ simply reflects that converted oxygen contributes nothing to O_2_ partial pressure—it is the natural reference point for the consumed flux.

The interstitial space constitutes a critical circuit node connecting oxygen delivery and consumption pathways. We measured interstitial oxygen partial pressure (*P*_i_) as the key system variable. We divided the circuit into two segments using the interstitial node: the delivery segment (arterial blood to interstitium, PO_2_ drop (*P*_a_ − *P*_i_)) and the consumption segment (interstitium to mitochondria, PO_2_ drop *P*_i_ − 0 = *P*_i_). Unless otherwise specified, PO_2_ in text refers to interstitial measurements in spinotrapezius muscle, with arterial values designated as *P*_a_. All measurements use mmHg units.

Oxygen flux (*V*) represents the electrical current analog, applicable to both extracellular delivery transit flow and intracellular consumption. We denote delivery oxygen flux as *V*_d_ and metabolic consumption rate VO_2_ as *V*_m_. For the two experimental states, oxygen consumption values are *V*_r_ (rest) and *V*_w_ (work). These parameters use units of nL O_2_/cm^3^ s.

Resistance factors limit oxygen transport, creating progressive PO_2_ decreases from aorta to mitochondria. Resistance relationships follow Ohm's law, determined by oxygen partial pressure differences and flux rates. Delivery resistance (*R*_d_) equals the PO_2_ difference between arterial and interstitial tensions (*P*_a_ − *P*_i_) divided by delivery flux (*V*_d_) (Equation 1). Metabolic pathway resistance is designated generally as *R*_m_, which takes specific values depending on the physiological state: *R*_r_ during rest and *R*_w_ during work. Resistance units are mmHg/(nL O_2_/cm^3^ s).

Oxygen capacitance represents tissue oxygen storage as capacitor C, relating locally stored oxygen concentration [O_2_] to interstitial fluid tension *P* (Equation 3). Capacitance units are nL O_2_/cm^3^ mmHg.

### 2.2 Model development from experimental data

Our electrical circuit model incorporated established experimental findings on oxygen delivery and consumption relationships in the rat spinotrapezius muscle. The respiratory circuit comprises sequential delivery and consumption components connected through the interstitial compartment, which functions as a natural voltage divider node for arterial blood oxygen tension.

We first determined oxygen consumption dependence on interstitial oxygen tension through direct measurements. Using oxygen probes loaded into microscopic muscle regions, we measured PO_2_ before and after rapid muscle compression that stopped blood flow and expelled microvascular blood. The resulting interstitial oxygen tension decrease created oxygen disappearance curves, which we differentiated and multiplied by the muscle Bunsen coefficient to establish the VO_2_(PO_2_) relationship (Golub & Pittman, 2012).

**FIGURE 1:**
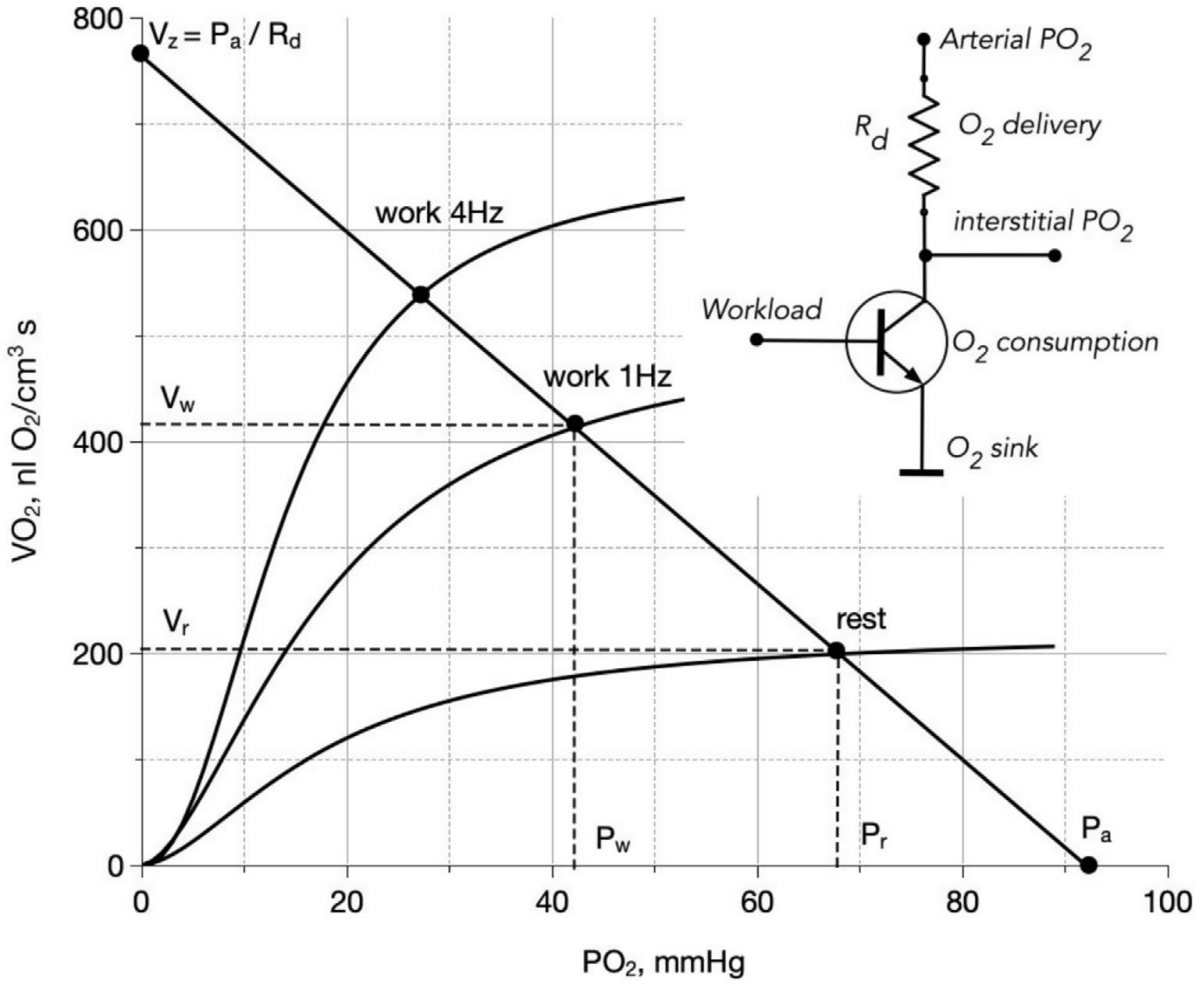
Electrical circuit analogy for oxygen delivery and consumption in skeletal muscle. The load line (diagonal solid line) represents oxygen delivery limitations with negative slope − 1/*R*_d_ indicating delivery resistance (Equation 1). Sigmoid curves demonstrate VO_2_ dependence on interstitial PO_2_ for different workloads, with higher curves corresponding to increased stimulation frequency. Intersection points determine steady‐state interstitial PO_2_ and the balance between oxygen delivery and consumption. Inset: Circuit schematic showing delivery resistance (*R*_d_) from arterial blood (*P*_a_) to interstitium (*P*_i_), and variable metabolic resistance (*R*_m_) from interstitium to mitochondria, represented as a transistor controlled by workload. Oxygen flux into and out of the interstitial space must be equal at steady state, allowing the transistor to be replaced by equivalent constant resistors (*R*_m_) for each workload condition.

In young resting muscles, VO_2_ dependence on PO_2_ proved nonlinear, steep at low PO_2_ levels and flatter at higher values. We subsequently established sigmoid curves for muscles pre‐stimulated with electrical impulses at 0.5, 1, 2, 4, and 8 Hz frequencies, with response magnitude directly correlating with workload (Golub et al., 2018). Figure 1 displays curves for rest, 1, and 4 Hz conditions, fitted using Hill equations. All curves demonstrate sigmoidal VO_2_ increases with rising interstitial PO_2_.

This workload‐dependent curve family resembles transistor current–voltage characteristics under varying base currents. In our circuit representation (Figure 1 inset), the oxygen consumption section appears as a common‐emitter transistor with workload‐proportional base control signals. When connected serially with delivery resistance, the VO_2_ (PO_2_) relationship generates a load line described by:

(1) $$V_{d}=\frac{\left(P_{a}-P_{i}\right)}{R_{d}}$$

This equation describes a straight line with a negative slope (−1/*R*_d_) intersecting the PO_2_ axis at point *P*_i_ = *P*_a_ (Figure 1). This line shows the limitations of interstitial *P*_i_ on the delivery part of system within the interstitial PO_2_ range from 0 to *P*_a_.

Each sigmoidal consumption curve operates within the same PO_2_ range. According to Kirchhoff's law for the interstitial node, steady‐state oxygen delivery and consumption fluxes must balance. This equilibrium occurs where the consumption sigmoid intersects the delivery line (Figure 1), providing a graphical solution to the system of two equations—one describing oxygen delivery and one describing oxygen consumption—that resolves the mathematical uncertainty of the interstitial PO_2_ value (*P*_i_). For any given workload level, cellular oxygen consumption rate is determined solely by the intersection point. For this point, we define the metabolic resistance (*R*_m_), that relates to interstitial PO_2_ and corresponding VO_2_ denoted as (*V*_m_):

(2) $$R_{m}=\frac{P_{i}}{V_{m}}$$

This approach simplifies the electrical analogy by replacing the transistor with a constant resistor for each workload level.

This representation of the balance O_2_ delivery and consumption is based on our PO_2_ and VO_2_ measurements in the interstitium of the spinotrapezius muscle (Golub et al., 2018). We found aligned the pre‐compression VO_2_ versus PO_2_ points for workloads produced by electric stimulation at 0.5, 1, 2, 4, and 8 Hz. These points fitted a straight line with a negative slope of *P*_d_ = 0.09 units of resistance. The delivery resistances calculated for individual pairs of PO_2_ and VO_2_ remained near constant in the range from 0.09 to 0.1 units. This finding indicated that VO_2_ was linearly limited by oxygen delivery to the muscle.

In other experiments, we measured interstitial PO_2_ during cycles of pneumatic compression (5 s) and decompression (15 s) to assess momentary VO_2_ values (Golub et al., 2023). The paired measurements of PO_2_ and VO_2_ were taken at rest, and during 1, 2, and 4 Hz electrical stimulation, as well as during recovery. The data indicated that the steady states for rest and work at 1–2 Hz stimulation align on the same line, intersecting the PO_2_ axis at the arterial PO_2_ point. The negative slope of this line was measured as *R*_d_ = 0.1 units. These data confirmed that *R*_d_ exhibited experimental constancy in young rat spinotrapezius muscle.

Compression measurements can distort the PO_2_ and VO_2_ transients time constants. Therefore, we designed subsequent experiments including rest, 1 Hz stimulation, and recovery to resting PO_2_ levels (Figure 3). We conducted this test on both young and old rats for comparative analysis of oxygen balance parameters in muscle at rest and during work, particularly regarding age‐related changes (Golub et al., 2025).

The foundational concepts and quantitative parameters of the electrical analogy model are based on experimental data. Further model development focuses on a single testable hypothesis regarding the crucial role of local oxygen storage in muscle in the forming PO_2_ transients.

### 2.3 Local oxygen storage modeling

**FIGURE 2:**
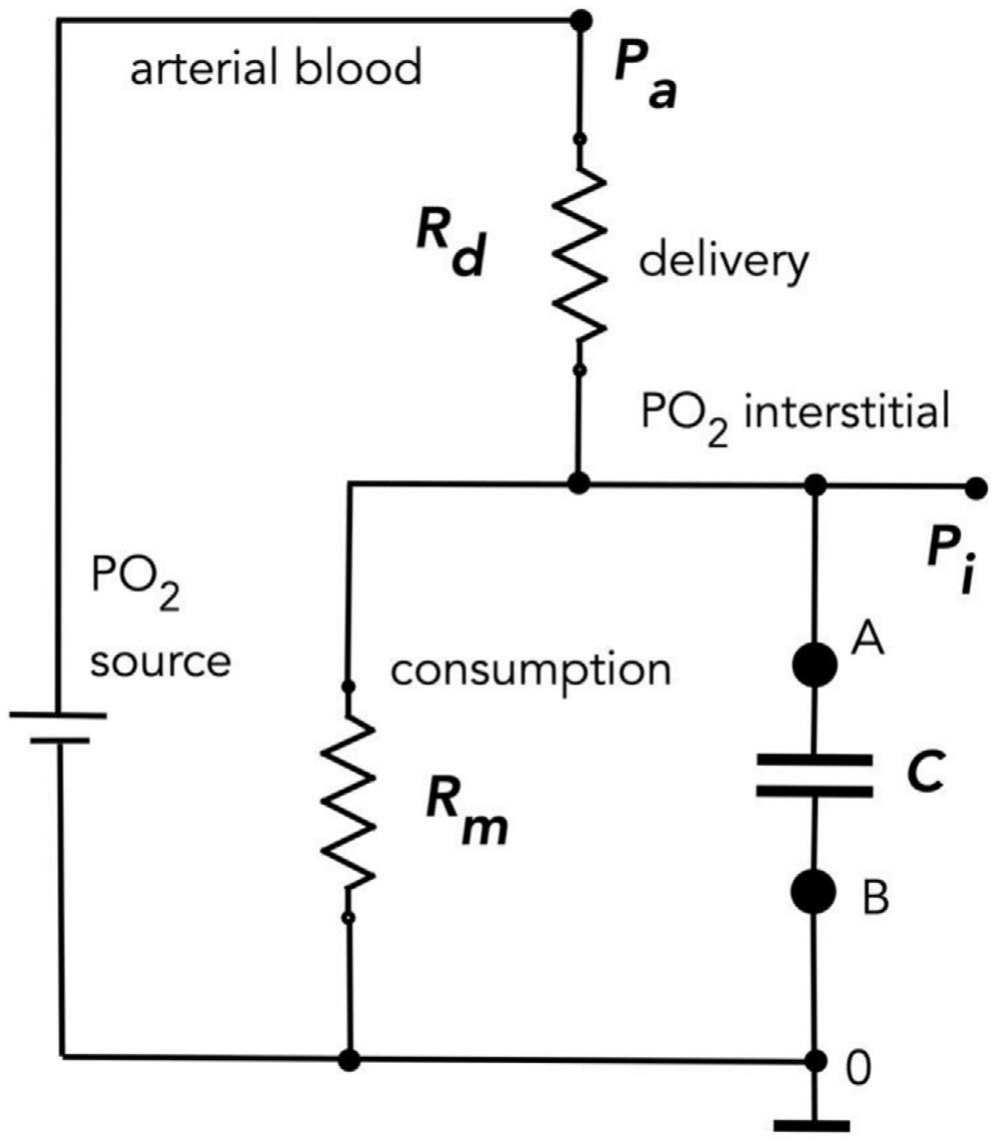
Complete electrical circuit model of oxygen transport and storage in skeletal muscle. The circuit incorporates arterial oxygen pressure (*P*_a_) as the voltage source, delivery resistance (*R*_d_), metabolic resistance (*R*_m_) that switches between resting (*R*_r_) and working (*R*_w_) values, and oxygen storage capacitance (*C*). Points A and B indicate measurement nodes for applying Thévenin's theorem analysis. This first‐order RC circuit configuration enables calculation of time constants governing interstitial PO_2_ transients during charging and discharging of muscle oxygen storage capacity, providing the theoretical foundation for predicting transition kinetics between rest and work states.

Comprehensive modeling requires accounting for dynamic rest‐work transitions beyond steady‐state conditions. We assume that at the transitions between rest and work, the VO_2_ rate in cells switches rapidly (~1 s) so that *R*_m_ takes constant values *R*_r_ or *R*_w_ without intermediate points. However, observed interstitial PO_2_ transitions exhibit exponential dynamics with time constants of several seconds. To explain this discrepancy, we introduce a capacitive element representing local oxygen storage in tissue. That oxygen storage appears as capacitor *C*, where the oxygen concentration [O_2_] related to its interstitial partial pressure, *P*_i_ (Figure 2):

(3) $$C=\frac{\left[O_{2}\right]}{P_{i}}$$

In steady state, capacitor *C* charges to be in asymptotic equilibrium with *P*_i_. When *P*_i_ decreases or increases, equilibrium disrupts and the capacitor discharges or charges through the equivalent resistor, *R*_e_, of the entire circuit, producing the exponential behavior observed experimentally. Capacitor discharging or charging through a resistor follows equations containing an exponential time course:

(4) e−tτ

where *t* is the time and *τ* is the time constant. For our analysis, the time constant *τ* equals:

(5) $$\tau =R_{e}\cdot C$$

where *R*_e_ is the equivalent resistor through which the capacitor *C* discharges or charges.

### 2.4 Circuit analysis using Thevenin's theorem

Our complete electrical circuit model (Figure 2) consists of a voltage source (*P*_a_), delivery resistance (*R*_d_), metabolic resistance (*R*_m_), and capacitor (*C*) representing oxygen storage. To analyze the transient responses of this first‐order RC circuit, we apply Thevenin's theorem.

First, we determine the steady‐state voltage ratio between points A and B with the capacitor disconnected using the voltage divider principle:

(6) $$\frac{P_{i}}{P_{a}}=\frac{R_{m}}{R_{d}+R_{m}}$$

In physiological terms, this refers to the ratio of interstitial PO_2_ to arterial blood PO_2_.

Next, we calculate the equivalent resistance (*R*_e_) seen by the capacitor. With the voltage source short‐circuited, *R*_e_ is given by the parallel combination of *R*_d_ and *R*_m_:

(7) $$R_{e}=\frac{R_{d}\cdot R_{m}}{R_{d}+R_{m}}$$

The time constant (*τ*) for charging or discharging of the capacitor through this equivalent resistance is:

(8) $$\tau =C\cdot \frac{R_{d}\cdot R_{m}}{R_{d}+R_{m}}$$

Substituting Equation (6) into Equation (8):

(9) $$\tau =C\cdot R_{d}\cdot \frac{P_{i}}{P_{a}}$$

where *P*_i_ is measured without capacitor *C* or in a steady state when capacitor *C* is inactive. This equation relates the time constant of PO_2_ transients to steady‐state interstitial PO_2_, providing a testable prediction of our model.

### 2.5 Rest‐work transition kinetics analysis

Since our experimental protocol involves transitions between distinct rest and work states, we can apply Equation 9 separately to each state. We denote steady‐state interstitial PO_2_ values as *P*_r_ for rest and *P*_w_ for work, with corresponding time constants *τ*_r_ and *τ*_w_.

The transition from rest to work occurs via rapid switching of metabolic resistance from *R*_r_ to *R*_w_ (where *R*_w_ < *R*_r_), while return to rest involves switching back to *R*_r_. The time course of interstitial PO_2_ during these transitions follows exponential curves with the time constants:

(10) $$\tau_{r}=C\cdot R_{d}\cdot \frac{P_{r}}{P_{a}};\tau_{w}=C\cdot R_{d}\cdot \frac{P_{w}}{P_{a}}$$

These equations predict that work‐to‐rest transitions should be slower than rest‐to‐work transitions because *P*_r_ > *P*_w_. This explains the asymmetry between onset and offset time constants, thus providing evidence for model validity. The relationship connects steady‐state PO_2_ values to transition speeds, as determined by the interaction of circuit elements.

Taking the ratio of these time constants:

(11) $$\frac{\tau_{r}}{\tau_{w}}=\frac{P_{r}}{P_{w}}$$

Equation (11) represents a critical testable prediction. If time constant ratios of PO_2_ work‐to‐rest and rest‐to‐work transitions essentially equal rest and work steady‐state PO_2_ value ratios, this would strongly support our hypothesis that exponential PO_2_ transients are governed by muscle oxygen storage processes rather than biochemical regulatory networks.

### 2.6 Resistance ratios and steady‐state oxygen flow calculations

Beyond transient kinetics, our model quantifies relative contributions of delivery and metabolic resistances during rest and work. These relationships derive from basic circuit configuration (Figure 2) with the capacitor removed, representing steady‐state conditions.

Metabolic resistances at rest and work relative to delivery resistance are:

(12) $$R_{w}/R_{d}=\frac{P_{w}}{\left(P_{a}-P_{w}\right)};R_{r}/R_{d}=\frac{P_{r}}{\left(P_{a}-P_{r}\right)}$$

From these expressions, we derive the ratio of metabolic resistances at rest and work:

(13) $$R_{r}/R_{w}=\frac{P_{r}\left(P_{a}-P_{w}\right)}{P_{w}\left(P_{a}-P_{r}\right)}$$

The overall oxygen flow resistance is (*R*_d_ + *R*_m_), which differs between rest (*R*_d_ + *R*_r_) and work (*R*_d_ + *R*_w_) states. According to Ohm's law, the ratio of VO_2_ rates between work and rest is inversely proportional to the ratio of these total resistances:

(14) $$\frac{V_{w}}{V_{r}}=\frac{R_{d}+R_{r}}{R_{d}+R_{w}}=\frac{1+R_{r}/R_{d}}{1+R_{w}/R_{d}};$$

These model equations provide frameworks for analyzing oxygen delivery and consumption changes using interstitial PO_2_ data alone. By inputting rest and exercise PO_2_ levels plus transient time constants, we calculate oxygen utilization characteristics in young muscle. Applying identical procedures to aged muscle parameters enables comparative analysis identifying age‐associated changes through differences in the calculated model components.

## 3 RESULTS

### 3.1 Experimental characterization of age‐related PO_2_ dynamics

We validated our electrical circuit model using experimental parameters from interstitial PO_2_ dynamics in the spinotrapezius muscle of rats during controlled exercise challenges. The study included both young (3‐month) and old (23‐month) Sprague–Dawley rats (Golub et al., 2025), with arterial blood PO_2_ data supplementing muscle measurements from the same cohort (Nugent et al., 2025).

**FIGURE 3:**
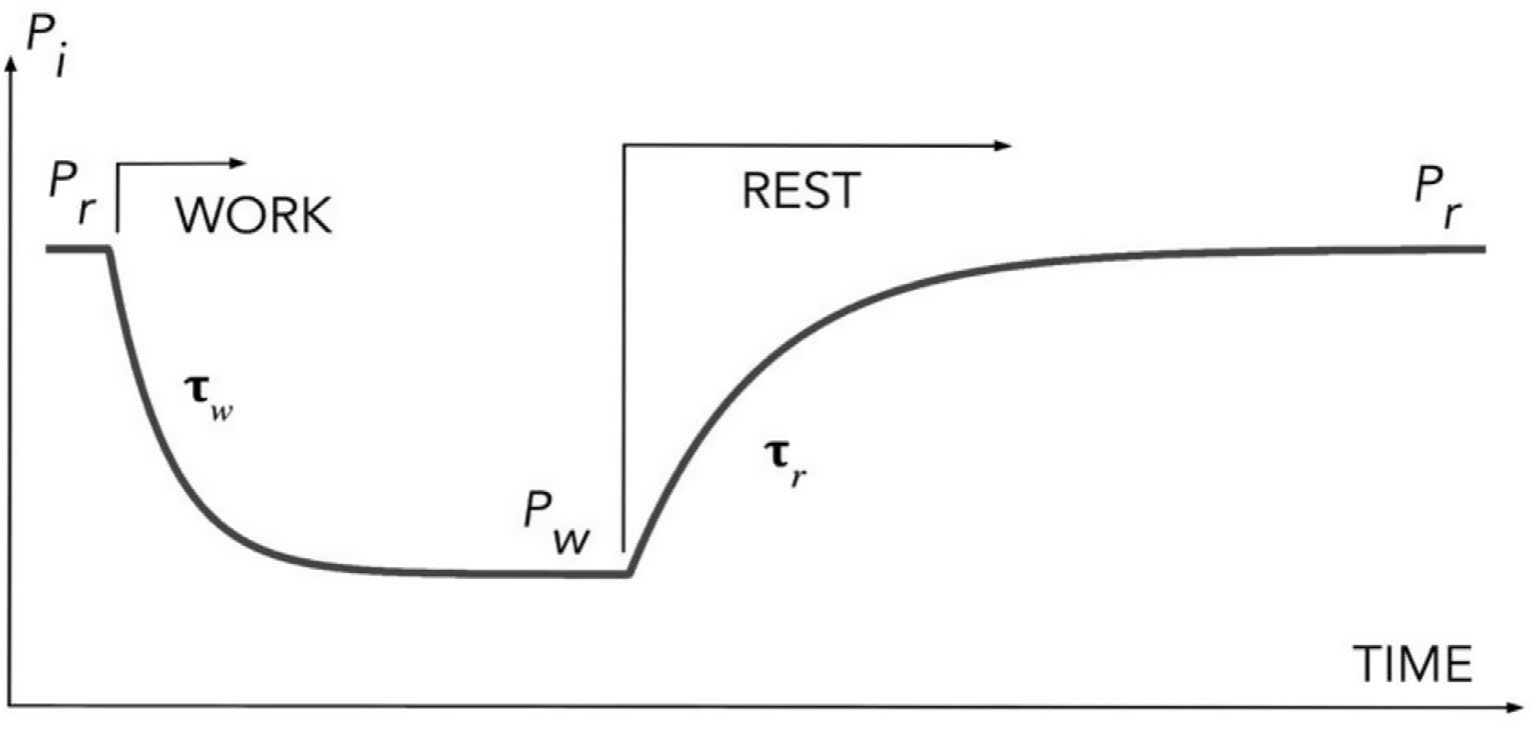
Experimental protocol and key parameters for rest‐to‐work and work‐to‐rest transitions. Representative time course of interstitial PO_2_ measurements in rat spinotrapezius muscle showing the experimental sequence: 10‐s baseline rest, 90 s of electrical stimulation at 1 Hz, and 150‐s recovery period (Golub et al., 2025). Steady‐state values *P*_r_ (rest) and *P*_w_ (work) represent baseline and working PO_2_ levels, respectively. Exponential transitions are characterized by time constants *τ*_w_ (rest‐to‐work) and *τ*_r_ (work‐to‐rest), with *τ*_r_ > *τ*_w_ demonstrating the asymmetric kinetics predicted by the electrical circuit model. This protocol enables quantification of both steady‐state oxygen balance and dynamic transition parameters for comparative analysis between young and aged muscle.

The experimental protocol (Figure 3) consisted of three sequential phases: 10‐s baseline recording at rest, 90 s of moderate‐intensity contractions induced by 1 Hz electrical stimulation, and a 150‐s recovery period enabling PO_2_ return to baseline levels. This design captured both rest‐to‐work and work‐to‐rest transitions within single experiments.

Table 1 summarizes experimental results revealing several notable age‐related differences. Old rats showed lower arterial PO_2_ (81.3 mmHg) compared to young rats (91.9 mmHg), representing an 11.5% reduction (Nugent et al., 2025). Baseline interstitial PO_2_ was lower in old muscles (60.2 mmHg) versus young muscles (66.7 mmHg), a 6.5 mmHg difference (Golub et al., 2025; McCullough et al., 2011; Russell et al., 2003). The decline in interstitial PO_2_ during muscle contraction was more pronounced in old muscles (28.3 mmHg) than young muscles (42.5 mmHg), widening the age‐related difference to 14.2 mmHg.

**TABLE 1:** Experimental parameters of oxygen transport in young and old rat spinotrapezius muscle.

Parameters	Symbol	Young rats	Old rats
Number of rats for blood PO_2_		11	12
Arterial blood PO_2_ ^a^	*P*_a_ (mmHg)	91.9 ± 5.4	81.3 ± 5.1
Number of interstitial PO_2_ profiles		117	139
Interstitial PO_2_ at rest	*P*_r_ (mmHg)	66.7 ± 2.5	60.2 ± 2.2
Interstitial PO_2_ at work	*P*_w_ (mmHg)	42.5 ± 2.6	28.3 ± 2.8
Rest‐to‐work time constant	τ_w_ (s)	9.0 ± 0.7	15.9 ± 0.6
Work‐to‐rest time constant	τ_r_ (s)	15.4 ± 0.7	41.4 ± 1.4

*Note*: Comprehensive characterization of oxygen dynamics during rest‐work‐rest transitions in young (3‐month) and old (23‐month) Sprague–Dawley rats. Arterial blood PO_2_ measurements establish systemic oxygen availability, while interstitial PO_2_ values at rest (*P*_r_) and during 1 Hz electrical stimulation (*P*_w_) quantify tissue‐level oxygen balance. Time constants for rest‐to‐work (*τ*_w_) and work‐to‐rest (*τ*_r_) transitions characterize the kinetics of oxygen storage mobilization. These parameters serve as primary inputs for electrical circuit model validation and enable quantitative comparison of age‐related changes in muscle oxygen transport mechanisms. Data represent Mean ± CI95% (95% confidence intervals).

^a^ Data for arterial blood PO_2_ were taken from (Nugent et al., 2025). Other data for interstitial PO_2_ were taken from (Golub et al., 2025).

The total PO_2_ difference from arterial blood to working muscle interstitium remained similar between young (49.4 mmHg) and old rats (53.0 mmHg), despite lower absolute values in old rats. Transition kinetics showed substantially longer time constants in old muscles (*τ*_w_ = 15.9 s, *τ*_r_ = 41.4 s) compared to young muscles (*τ*_w_ = 9.0 s, *τ*_r_ = 15.4 s), indicating slower oxygen dynamics with age.

Both age groups displayed the asymmetry in transition kinetics predicted by our model, with work‐to‐rest transitions (*τ*_r_) occurring more slowly than rest‐to‐work transitions (*τ*_w_).

### 3.2 Model prediction validation

A critical test of our model's validity is the relationship expressed in Equation (11), which predicts that the ratio of time constants (*τ*_r_/*τ*_w_) should equal the ratio of steady‐state PO_2_ values (*P*_r_/*P*_w_). This prediction stems from our hypothesis that interstitial PO_2_ transients are governed primarily by processes of discharging and charging the muscle oxygen storage.

We calculated these ratios from our experimental data (Table 2). For young muscles, the ratio of time constants (*τ*_r_/*τ*_w_ = 1.7) closely matched the ratio of steady‐state PO_2_ values (*P*_r_/*P*_w_ = 1.6). This agreement supports our model and its underlying assumptions.

**TABLE 2:** Circuit model parameters calculated for young and old muscles.

Ratios	Symbolic	Young muscles	Old muscles
Time constants, rest/work	*τ*_r_/*τ*_w_	1.7	2.6
Interstitial PO_2_'s, rest/work	*P*_r_/*P*_w_	1.6	2.1
Resistances, rest/delivery	*R*_r_/*R*_d_	2.6	2.8
Resistances, work/delivery	*R*_w_/*R*_d_	0.86	0.53
Resistances, rest/work	*R*_r_/*R*_w_	3.1	5.4
O_2_ consumption, work/rest	*V*_w_/*V*_r_	2.0	2.5

*Note*: Dimensionless ratios derived from electrical circuit analysis reveal functional relationships between oxygen transport components. Time constant ratios (*τ*_r_/*τ*_w_) test the fundamental model prediction that transition kinetics reflect oxygen storage processes rather than biochemical regulation. Resistance ratios quantify the relative contributions of delivery limitations (*R*_d_) versus metabolic regulation (*R*_m_) during rest and work states. The dramatic decrease in metabolic resistance from rest to work (*R*_r_/*R*_w_ ratios of 3.1 and 5.4 for young and old muscle, respectively) demonstrates preserved regulatory capacity in aging muscle. Oxygen consumption ratios (*V*_w_/*V*_r_) indicate the functional reserve available during activity transitions.

For old muscles, the agreement was less precise but still supportive. The time constants ratio (*τ*_r_/*τ*_w_ = 2.6) exceeded the steady‐state PO_2_ ratio (*P*_r_/*P*_w_ = 2.1) by 19%. This moderate discrepancy suggests that while the same physical principles apply in aged muscle, additional factors may slightly modify the relationship. Notable is the significant increase in resistances in aged muscles (Table 3). Possible explanations include age‐related changes in tissue composition affecting oxygen solubility or subtle differences in the spatial distribution of oxygen‐consuming mitochondria.

**TABLE 3:** Comparative analysis of model parameters between old and young muscles.

Ratios	Old (1)/young (2)	–
Arterial blood PO_2_'s	*P*_a2_/*P*_a1_	0.88
Delivery resistances	*R*_d2_/*R*_d1_	2.5
Metabolic resistances at rest	*R*_r2_/*R*_r1_	2.7
Metabolic resistances at work	*R*_w2_/*R*_w1_	1.5
Overall resistances at rest	(*R*_d2_ + *R*_r2_)/(*R*_d1_ + *R*_r1_)	2.6
Overall resistances at work	(*R*_d2_ + *R*_w2_)/(*R*_d1_ + *R*_w1_)	2.1
O_2_ consumptions at rest	*V*_r2_/*V*_r1_	0.33
O_2_ consumptions at work	*V*_w2_/*V*_w1_	0.43

*Note*: Quantitative assessment of age‐related changes expressed as ratios of old‐to‐young muscle parameters. The second index stands for the indicated age: 1 for young and 2 for old muscles. The 2.5‐fold increase in delivery resistance (*R*_d2_/*R*_d1_) indicates substantial microvascular impairment with aging, while metabolic resistance changes reveal complex adaptations: higher resting values (2.7‐fold) but smaller differences during work (1.5‐fold). Combined resistance effects result in reduced absolute oxygen consumption in old muscle, yet the relative improvement from rest to work (0.33–0.43) demonstrates significant functional reserve. These ratios provide a quantitative framework for understanding how aging affects the balance between oxygen delivery limitations and metabolic adaptations in skeletal muscle.

The experimental data support our model's core prediction and the hypothesis that interstitial PO_2_ transients during activity transitions are primarily shaped by local oxygen storage mobilization.

### 3.3 Resistance component analysis in age‐related changes

Having validated the model's basic assumptions, we next quantified specific resistance components in young and old muscles to gain insight into age‐related functional adaptations. Using Equations ((12), (13), (14)) and the experimental parameters from Table 1, we calculated the relative resistances to oxygen transport and consumption (Table 2).

In young muscles at rest, metabolic resistance (*R*_r_) was 2.6 times greater than delivery resistance (*R*_d_), indicating that VO_2_ at rest was primarily limited by metabolic factors rather than by oxygen supply. During work this relationship reversed, with metabolic resistance decreasing to only 0.86 times the delivery resistance. This 3.1‐fold reduction in metabolic resistance at work illustrates the young muscle's ability to regulate oxygen utilization by shifting the limiting factor between metabolism and delivery.

Old muscles showed both similarities and differences in this pattern. At rest, old muscles displayed a similar metabolic to delivery resistance ratio (*R*_r_/*R*_d_ = 2.8). During contraction, metabolic resistance decreased to 0.53 times the delivery resistance, representing a 5.4‐fold reduction from the resting value—almost double the relative change observed in young muscles.

This more pronounced metabolic resistance reduction enabled old muscles to increase VO_2_ during exercise by 2.5‐fold compared to rest, which exceeded the 2.0‐fold increase observed in young muscles. These findings suggest that while aged muscles operate at lower absolute VO_2_ values (Table 3), they retain substantial capacity to regulate metabolic resistance and oxygen utilization during rest‐to‐work transitions.

### 3.4 Quantitative age‐related parameter differences

To directly compare oxygen transport characteristics between young and old muscles, we established conversion relationships between model instantiations. We denoted parameters for young rats with subscript 1 and old rats with subscript 2, using the delivery resistance in young muscle (*R*_d1_) as reference.

We conducted independent calculations to compare delivery resistances based on resting and working state data. From Equation (10) we derived expressions relating the delivery resistances in young and old muscles at rest and work:

(15) $$\frac{R_{d2}}{R_{d1}}=\frac{\tau_{r2}\cdot P_{r1}\cdot P_{a2}}{\tau_{r1}\cdot P_{r2}\cdot P_{a1}};\frac{R_{d2}}{R_{d1}}=\frac{\tau_{w2}\cdot P_{w1}\cdot P_{a2}}{\tau_{w1}\cdot P_{w2}\cdot P_{a1}}$$

Calculations yielded two values of ratios of 2.6 and 2.4. Close agreement between these two results validates our model's internal consistency. For subsequent calculations, we used the average value of *R*_d2_/*R*_d1_ = 2.5.

By applying *R*_d2_ = 2.5 · *R*_d1_ substitutions to Ohm's law for the circuit in Figure 2, we calculated VO_2_ ratios in steady states of work and rest for old versus young muscles (Table 3). This represents arterial PO_2_ ratios in old and young rats multiplied by their total resistance ratios at rest and work:

(16) $$\frac{V_{r2}}{V_{r1}}=\frac{P_{a2}}{P_{a1}}\cdot \frac{R_{d1}+R_{r1}}{R_{d2}+R_{r2}};\frac{V_{w2}}{V_{w1}}=\frac{P_{a2}}{P_{a1}}\cdot \frac{R_{d1}+R_{w1}}{R_{d2}+R_{w2}}$$

Using this conversion factor and previously calculated resistance ratios (Table 2), we determined comparative relationships between all model parameters for young and old muscles (Table 3). These calculations revealed several significant age‐related differences.

The 2.5‐fold increase in delivery resistance (*R*_d2_/*R*_d1_ = 2.5) represents substantial impairment in oxygen transport from blood to the interstitial space in aged muscle. This change likely reflects alterations in microvascular structure and function with aging.

At rest, metabolic resistance was 2.7 times higher in old muscle compared to young muscle (*R*_r2_/*R*_r1_ = 2.7), consistent with reduced basal metabolic activity. During work, this difference was substantially smaller (*R*_w2_/*R*_w1_ = 1.5), reflecting the robust capacity of aged muscle to activate VO_2_ during exercise.

The combined effect of delivery and metabolic resistances resulted in substantially higher total resistance in old muscle, both at rest (2.6‐fold higher) and during work (2.1‐fold higher). This differential explains the reduced absolute VO_2_ rates in aged muscle.

Due to both increased resistance and slightly lower arterial PO_2_, VO_2_ in old muscle was only 33% of that in young muscle at rest (*V*_r2_/*V*_r1_ = 0.33). During contraction, this ratio improved to 43% (*V*_w2_/*V*_w1_ = 0.43), demonstrating that old muscle retains greater relative functional capacity during activity than might be expected from resting measurements alone.

These quantitative comparisons provide insights into specific adaptations and limitations characterizing aging skeletal muscle, showing that despite substantial increases in both delivery and metabolic resistances, old muscle maintains significant capacity to regulate VO_2_ during activity transitions.

## 4 DISCUSSION

### 4.1 Physiological implications of the circuit model

The electrical circuit model provides a powerful tool for visualizing and simulating oxygen transport and utilization dynamics in skeletal muscle. It captures the self‐regulating nature of oxygen transport in muscle. The interaction between passive delivery resistance (*R*_d_) and actively regulated metabolic resistance (*R*_m_) automatically adjusts oxygen flow to match demand through changes in the (*P*_a_ − *P*_i_) difference (Figure 1). This mechanism explains how skeletal muscle can rapidly increase oxygen consumption (VO_2_) without immediate proportional increases in blood flow or other delivery parameters.

The model clarifies why metabolic regulation of VO_2_ is more efficient than vascular regulation. When metabolic resistance decreases during exercise, the operating point shifts to a higher position on delivery line, increasing oxygen flux due to greater PO_2_ differences between microvascular and interstitial compartments. In contrast, decreasing delivery resistance would shift the operating point toward the flatter portion of the consumption curve, yielding a smaller oxygen flow increase.

By incorporating a capacitive element, the circuit model provides a physical basis for the exponential transients observed during activity transitions (Figure 2). This approach resolves debates about whether these transients reflect gradual mitochondrial network activation or simple physical processes. The model explained transient asymmetry between rest‐to‐work and work‐to‐rest time constants and predicted their connection to interstitial PO_2_ values in these states. Experimental data demonstrated that the ratio of time constants closely matches the ratio of steady‐state PO_2_ value ratios at rest and work, strongly supporting the tissue oxygen capacitance mechanism.

### 4.2 Age‐related changes in oxygen transport and utilization

Comparison of parameters calculated for young and old muscles reveals significant age‐related alterations in oxygen transport and utilization (Table 3). The 2.5‐fold higher delivery resistance (*R*_d_) in old muscle indicates a substantial impairment in oxygen transport from blood to the interstitial space. This transport decline may be attributed to reduced capillary network density and increased diffusion distances due to muscle fiber diameter enlargement in aging muscles.

Age‐related changes in metabolic resistance present a more complex picture. The 2.7‐fold increase in resting metabolic resistance (*R*_r_) in old muscle is consistent with decreased basal metabolic activity, potentially reflecting mitochondrial dysfunction, reduced mitochondrial density, or shifts in fiber type composition (Table 3). However, the ability of old muscle to decrease metabolic resistance 5.4‐fold during exercise (compared to 3.1‐fold in young muscle) demonstrates functional reserve in old muscles (Table 2).

### 4.3 Relationship to established aging theories

Our findings contribute quantitative insights to the ongoing debate between vascular and mitochondrial hypotheses of muscle aging. The substantial increase in oxygen delivery resistance with age supports the vascular mechanism, indicating that impaired oxygen transport constrains functioning of older muscle (Ungvari et al., 2018). This finding aligns with previous observations of decreased microvascular PO_2_ and slower oxygen kinetics in aged muscle (McCullough et al., 2011; Russell et al., 2003).

However, our data also relate to aspects of the mitochondrial theory. The reduced VO_2_ at rest (only 33% of young muscle values) is consistent with decreased mitochondrial content or efficiency. Yet, the capacity to decrease metabolic resistance during exercise challenges the notion of intrinsic mitochondrial dysfunction as a primary limitation. Instead, our results suggest that while overall mitochondrial content may be reduced, the remaining mitochondria in old muscle retain significant functional capacity.

A synthesis of these observations suggests that both vascular and cellular factors contribute to age‐related changes in muscle energetics, but their relative importance differs between rest and exercise states. At rest, both factors appear to limit VO_2_, while during exercise, vascular limitations become predominant as old muscle demonstrates substantial capacity to activate mitochondrial respiration.

### 4.4 Sarcopenia and fiber type composition effects

The seemingly paradoxical finding that old muscle can decrease metabolic resistance more dramatically during exercise may be explained by age‐related changes in muscle fiber composition associated with sarcopenia (Larsson et al., 2019). Published data demonstrate that mitochondrial respiration does not deteriorate with aging (Distefano et al., 2017; Rasmussen et al., 2003a, 2003b). It can be inferred that the capacity for compensatory reactions in aging muscles reflects the overall quantity of mitochondria present in the cells. Changes in muscle composition due to sarcopenia can account for the observed phenomenon.

Aging preferentially affects fast‐twitch glycolytic (type II) fibers, with relative preservation of slow‐twitch oxidative (type I) fibers (Dao et al., 2020; Demontis et al., 2013; Marzetti et al., 2008). This selective atrophy results in a “fast‐to‐slow shift” in the overall muscle fiber profile (Dowling et al., 2023). Since type I fibers contain higher mitochondrial density and oxidative capacity, this compositional shift may increase mitochondrial concentration in remaining muscle tissue of aged animals. The adult rat spinotrapezius muscle normally contains approximately 32% type I fibers (Taylor & Calvey, 1977), but this percentage likely increases with age due to preferential loss of type II fibers.

This explanation reconciles our findings with previous studies reporting both decreased absolute VO_2_ capacity and increased specific VO_2_ in aging muscle (Hepple et al., 2003). While total mitochondrial content decreases with muscle atrophy, mitochondrial density in remaining fibers may increase, enhancing the specific capacity for oxidative metabolism. This would explain the greater relative increase in VO_2_ from rest‐to‐work in old muscle (2.5‐fold) compared to young muscle (2.0‐fold).

### 4.5 Model limitations

Several important limitations should be considered when interpreting our results.

The model was designed and validated using rat spinotrapezius muscle data. While this thin, planar muscle provides an excellent model for studying microcirculation principles, it differs from typical locomotor muscles in fiber composition and mechanical function. Future studies should apply these experimental and analytical principles to muscles directly involved in locomotion, where controlling and measuring power output during loading becomes essential.

The circuit model is conceptual and currently limited to moderate exercise power and two discrete steady states to avoid nonlinear effects in circuit components. This constraint could be addressed by implementing the model in circuit simulation software capable of handling dynamic conditions beyond low‐intensity exercise.

The model validation relies on a single experimental protocol—1 Hz stimulation for 90 s in one muscle type. Broader validation across different exercise intensities, durations, and muscle types is needed to establish the model's general applicability to skeletal muscle oxygen transport.

### 4.6 Compensatory adaptations in aging skeletal muscle

The ability of old muscle to decrease metabolic resistance by 5.4‐fold during exercise (compared to 3.1‐fold in young muscle) represents a powerful adaptive mechanism that partially offsets increased delivery resistance. Functionally, aging muscle can achieve 43% of young muscle VO_2_ during exercise, despite consuming only 33% at rest. This finding indicates that aging muscle retains greater reserve capacity than might be predicted from resting measurements alone, which has important implications for understanding exercise tolerance and designing interventions to maintain physical function in older individuals.

The physiological basis for this enhanced metabolic regulation likely involves both cellular and systemic adaptations. At the cellular level, the shift toward oxidative fiber types increases mitochondrial density in remaining muscle tissue. At the systemic level, the lower resting VO_2_ may represent an energy‐conserving adaptation that preserves resources for periods of activity.

These findings challenge the common perception of aging muscle as uniformly dysfunctional and highlight sophisticated adaptive mechanisms that operate to maintain essential physiological functions throughout the lifespan. Rather than simple deterioration, aging muscle undergoes complex remodeling that sacrifices certain capabilities (absolute power and speed) to preserve others (endurance and efficiency).

## 5 CONCLUSION

The electrical circuit model provides a quantitative method for analyzing oxygen transport and utilization in skeletal muscle across the lifespan. The model demonstrates how the interaction between passive delivery resistance and actively regulated metabolic resistance automatically establishes new steady‐state interstitial PO_2_ values during rest‐work transitions through voltage divider principles. The model's prediction that interstitial PO_2_ transition kinetics are determined by the interaction of tissue O_2_ capacitance with circuit resistances was supported by experimental validation, with time constant ratios matching steady‐state PO_2_ ratios as predicted.

Aged muscle exhibits 2.5‐fold higher O_2_ delivery resistance, yet retains remarkable capability to regulate metabolic resistance during activity transitions—decreasing it 5.4‐fold during exercise compared to 3.1‐fold in young muscle. This enhanced metabolic regulation represents a sophisticated compensatory mechanism that partially offsets age‐related vascular limitations. Aging muscle undergoes complex remodeling that preserves essential regulatory capabilities despite structural constraints.

## AUTHOR CONTRIBUTIONS

Conception and research design: A.S.G., R.N.P., B.K.S., and W.H.N. Interpretation of results: A.S.G. and R.N.P. Manuscript and figures preparation: A.S.G., R.N.P., B.K.S., and W.H.N. Editing and revising manuscript: A.S.G., R.N.P., B.K.S., and W.H.N. Final version of MS approval: A.S.G., R.N.P., B.K.S., and W.H.N.

## FUNDING INFORMATION

This study was funded and performed by Song Biotechnologies, LLC.

## CONFLICT OF INTEREST STATEMENT

Aleksander Golub, William Nugent, and Bjorn Song are employees of Song Biotechnologies, LLC, which funded this research. However, the company had no direct role in the design, conduct, analysis, or interpretation of the study and was not involved in the preparation of the manuscript. The research design, data analysis, and manuscript preparation were conducted independently by the scientific team. Roland Pittman received no compensation for his contributions and has no conflicts of interest to declare.

## ETHICS STATEMENT

Experimental data were obtained from literature sources. No subjects were used to devlop this theoretical model.

## Data Availability

For original data, please contact bjorn@songbiotechnologies.com.
